# Upregulated Nuclear Expression of Soluble Epoxide Hydrolase Predicts Poor Outcome in Breast Cancer Patients: Importance of the Digital Pathology Approach

**DOI:** 10.3390/ijms25158024

**Published:** 2024-07-23

**Authors:** Mayra Montecillo-Aguado, Giovanny Soca-Chafre, Gabriela Antonio-Andres, Mario Morales-Martinez, Belen Tirado-Rodriguez, Angelica G. Rocha-Lopez, Daniel Hernandez-Cueto, Sandra G. Sánchez-Ceja, Berenice Alcala-Mota-Velazco, Anel Gomez-Garcia, Sergio Gutiérrez-Castellanos, Sara Huerta-Yepez

**Affiliations:** 1 Unidad de Investigación en Enfermedades Oncológicas, Hospital Infantil de México, Federico Gómez, Ciudad de México 06720, Mexico; mayramontecillo@gmail.com (M.M.-A.); g.s.ch@outlook.com (G.S.-C.); gabya_24@yahoo.com.mx (G.A.-A.);; 2 División de Estudios de Posgrado, Facultad de Ciencias Médicas y Biológicas Dr. Ignacio Chávez, Universidad Michoacana de San Nicolás de Hidalgo (UMSNH), Morelia 58060, Mexico; 3 Laboratorio de Patología, Facultad de Químico Farmacobiología, Universidad Michoacana de San Nicolás de Hidalgo (UMSNH), Morelia 58060, Mexico; sgsanchezc@gmail.com; 4 Departamento de Patología, Facultad de Odontología, Universidad Michoacana de San Nicolás de Hidalgo (UMSNH), Morelia 58060, Mexico; bamota@umich.mx; 5 Centro de investigación Biomédica de Michoacán, División de Investigación Clínica, Instituto Mexicano del Seguro Social, Morelia 58060, Mexico; anel.gomez@imss.gob.mx

**Keywords:** breast cancer (BC), soluble epoxide hydrolase (EPHX2), overall survival (OS), digital pathology (DP), tissue microarray (TMA)

## Abstract

Breast cancer (BC) is the most common cancer in women, with incidence rates increasing globally in recent years. Therefore, it is important to find new molecules with prognostic and therapeutic value to improve therapeutic response and quality of life. The polyunsaturated fatty acids (PUFAs) metabolic pathway participates in various physiological processes, as well as in the development of malignancies. Although aberrancies in the PUFAs metabolic pathway have been implicated in carcinogenesis, the functional and clinical relevance of this pathway has not been well explored in BC. To evaluate the clinical significance of soluble epoxide hydrolase (EPHX2) expression in Mexican patients with BC using tissue microarrays (TMAs) and digital pathology (DP). Immunohistochemical analyses were performed on 11 TMAs with 267 BC samples to quantify this enzyme. Using DP, EPHX2 protein expression was evaluated solely in tumor areas. The association of EPHX2 with overall survival (OS) was detected through bioinformatic analysis in public databases and confirmed in our cohort via Cox regression analysis. Clear nuclear expression of EPHX2 was identified. Receiver operating characteristics (ROC) curves revealed the optimal cutoff point at 2.847062 × 10^−3^ pixels, with sensitivity of 69.2% and specificity of 67%. Stratification based on this cutoff value showed elevated EPHX2 expression in multiple clinicopathological features, including older age and nuclear grade, human epidermal growth factor receptor 2 (HER2) and triple negative breast cancer (TNBC) subtypes, and recurrence. Kaplan–Meier curves demonstrated how higher nuclear expression of EPHX2 predicts shorter OS. Consistently, multivariate analysis confirmed EPHX2 as an independent predictor of OS, with a hazard ratio (HR) of 3.483 and a 95% confidence interval of 1.804–6.724 (*p* < 0.001). Our study demonstrates for the first time that nuclear overexpression of EPHX2 is a predictor of poor prognosis in BC patients. The DP approach was instrumental in identifying this significant association. Our study provides valuable insights into the potential clinical utility of EPHX2 as a prognostic biomarker and therapeutic target in BC.

## 1. Introduction

Breast cancer (BC) is the most common type of cancer globally, with 2.2 million new cases in 2020. It ranks fifth in overall cancer mortality, being the most frequent neoplasia in women. In addition to the above, it is estimated that 684,996 deaths worldwide were due to BC; thus, it is becoming an important public health issue [1] BC is usually classified into four subtypes according to receptor expression: luminal A, luminal B, human epidermal growth factor receptor 2 (HER2+) overexpression, and basal (triple negative breast cancer, (TNBC)) [2]. Clinical evolution differs across subtypes and therefore so does the treatment, together with other clinicopathological characteristics. Luminal subtypes are characterized by the expression of hormonal receptors (HR), and they are considered less aggressive; those overexpressing HER2 display more aggressive behavior; and TNBC show the worst prognosis due to their biological characteristics and fewer therapeutic options compared to other subtypes [3,4]. Deeper knowledge of tumor behavior and the discovery of new biomarkers allowed for the identification of various prognostic patterns in BC. Despite the growing number of clinically relevant molecular subtypes, current management of BC patients still relies on traditional pathological evaluation. On the other hand, clinical stage is a key element guiding therapeutic conduct, as well as prognostic factors, since patients behave differently according to stage [5,6]. Therefore, it is important to search for new molecular biomarkers to better support diagnosis and treatment.

In this sense, experimental and epidemiological evidence supports the participation of omega-3 and omega-6 polyunsaturated fatty acids (PUFAs), as well as their oxylipins in cancer development [7]. One of the main pathways responsible for the PUFAs metabolism involves the CYP450/soluble epoxide hydrolase (EPHX2) arm [8]. Therefore, the balance between cytochromes and EPHX2 expression is extremely important and responsible for sustaining the concentration of epoxides as highly reactive metabolites. In particular, EPHX2 enzyme catalyzes the hydroxylation of active epoxides to their corresponding dihydrodiols (fewer active metabolites) by adding a water molecule [9]. EPHX2 is located mainly in cytosol and in peroxisomes, as well as in the nucleus. It is a homodimer composed of two 62 kDa monomers. The C-terminal domain contains the active hydrolase site, consisting of a catalytic domain containing two tyrosine residues (Tyr381 and tyr564), which activate the epoxide ring, opening via Asp333, and the resulting ester is rapidly hydrolyzed to dihydrodiol. In addition, the N-terminal domain presents the phosphatase active site dependent on magnesium [10,11].

Several authors have found differential expressions of this enzyme, depending on cancer type. For example, Enayetallah et al. reported the loss of EPHX2 expression in renal and hepatic carcinoma compared to normal tissue. On the other hand, they also reported upregulated EPHX2 expression in cholangiocarcinoma, testicular seminoma, and colon adenocarcinoma compared to non-cancerous tissue. Likewise, all patients with stage 3 ovarian adenocarcinoma had EPHX2 staining in contrast to normal tissue and stages 1–2; these data suggest an oncogenic role [12]. On the other hand, Panigrahy et al. showed reduced EPHX2 protein expression in tumor tissue in a murine model of Lewis lung carcinoma. In the same study, the authors also found lower EPHX2 levels in melanoma-derived liver metastases compared to healthy tissue [13]. Particularly, in BC, some in silico assays have reported EPHX2 activity. For example, Yue et al. identified EPHX2 as potential prognostic and predictive molecular markers for clinical outcome in BC patients [14]. In addition, Thomassen et al., through a meta-analysis, identified EPHX2 as a candidate for the metastasis-suppressor gene in BC [15]. Also, Wang et al., through transcriptome-wide analysis and modeling of prognostic alternative splicing signatures in invasive BC, followed by qRT-PCR in TNBC patients, found exon-specific EPHX2 expression associated with patient status under neoadjuvant chemotherapy [16]. On the other hand, an in vitro assay in MDA-MB-231 cells showed how EPHX2 overexpression through an adenoviral vector reduced cellular proliferation and migration [17]. In the same study, Wei et al. found lower EPHX2 protein levels in BC vs. normal tissue. Likewise, EPHX2 expression negatively correlated with tumor size, estrogen receptors, and Ki67 expression [17]. Moreover, MCF7 cells treated with low fibrate doses (PARα ligand) stimulated cell proliferation, accompanied by increased CYP2C8 levels and lower EPHX2 expression [18]. All this in vitro evidence suggests a suppressor role for this enzyme. Additionally, Enayetallah et al., through semi-quantitative evaluation of staining intensity, reported that 60% of patients with breast ductal adenocarcinoma had moderate EPHX2 staining versus control tissue [12]. Taken together, these results imply that EPHX2 could play either suppressor or oncogene roles, depending on the cancer type. Despite many studies characterizing and supporting suppressor EPHX2 activity in BC, the participation of this enzyme remains poorly understood. To this end, we examined EPHX2 expression in a cohort of BC patients through tissue microarrays (TMAs) and digital pathology (DP).

## 2. Results

### 2.1. Patient Cohort and Clinicopathological Characteristics

We retrospectively analyzed clinical data from 267 patients diagnosed with primary invasive BC between 2015 and 2017 (Table 1). Women were stratified into low and high EPHX2 levels to contrast with their clinicopathological characteristics according to the value of 2.847062 × 10^−3^ pixels described in the section of biomarker cutoff below. Multiple variables were dichotomized to detect meaningful relationships with EPHX2. In this cohort, 234 (87.6%) and 33 (12.4%) patients with high and low EPHX2 expression were identified, respectively. Overall age ranged from 25 to 86 years, with a median of 52 years. Age groups included mostly middle-aged women (45–59 yrs) (47.2%) and older (≥60 yrs) (30.3%), while adult women (26–44 yrs) accounted for 22.1% and the young category (18–25 yrs) for only one case (0.35%). Invasive ductal carcinoma was the major histological type, with 238 cases (89.1%); minor types included infiltrating lobular carcinoma (4.5%), mucinous carcinoma (1.9%), ductal carcinoma in situ (0.7%), metaplastic carcinoma (1.1%), and papillary and medullary carcinomas at 0.4% and 0.7%, respectively, whereas pleomorphic, adenoid cystic, neuroendocrine, and mixed carcinomas represented 0.4% each. Most patients presented intermediate histologic and nuclear grades. Morphologic analysis also showed that the non-infiltrative border was more abundant in this cohort (75.7%), as well as tumors of larger diameter >2 cm (57.7%). Axillary lymph node dissection or lymphadenectomy was performed in approximately two thirds of cases. Women were predominantly estrogen (68.2%) and progesterone (58.1%) receptors-positive (ER+, PR+), but negative (82.6%) for human epidermal growth factor receptor 2 (HER2−). According to molecular subtype, luminal A and B were the most frequent, accounting for almost 70% of patients, while TNBC was 20% and HER2 only 10%. American Joint Committee on Cancer (AJCC) prognostic stage showed predominance in the early stages, which combined (I + II) represented 75% of the cohort, and similarly, more than three quarters of patients had no recurrence. Table 1 shows that EPHX2 expression differed significantly in some categories of clinicopathological variables, as measured via Chi-square and Fisher´s exact tests; for example, elevated levels of this enzyme were associated with non-infiltrative border type (*p* = 0.030) and smaller tumor size (*p* = 0.090). The percentage of high EPHX2 expression was 90.9% in non-infiltrative vs. only 9.1% in infiltrative borders. In the same manner, high EPHX2 values were more frequent in patients with tumors ≤2 cm (57.6%) vs. those with tumor diameter >2 cm (42.4%). Moreover, Supplementary Figure S1 shows numeric associations between EPHX2 and patient features, measured via Mann–Whitney U test, given the non-normal distribution of expression data. EPHX2 level was higher in patients ≥52 years old, with nuclear grade 3, HER2+ or TNBC subtype, and recurrent disease; conversely, EPHX2 expression was lower in ER+ women.

### 2.2. Nuclear EPHX2 Expression in BC and Digital Pathology Approach

**Figure 1:**
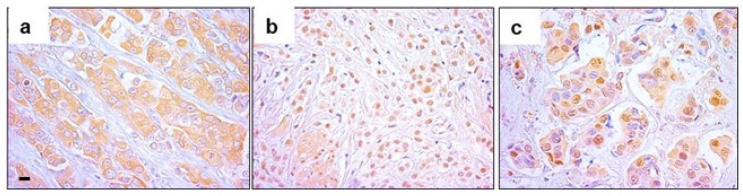
Differential expression of EPHX2 in the tumor tissue of breast cancer patients. EPHX2 expression levels were evaluated using immunohistochemistry staining (IHC) in (**a**) cytoplasmic expression of EPHX2, (**b**) nuclear expression of EPHX2, and (**c**) expression of EPHX2, both in cytoplasm and the nucleus. Scale bar = 10 μm.

We investigated EPHX2 protein expression in a cohort of BC patients. For this purpose, we performed immunohistochemistry (IHC) analysis on a TMAs consisting of 267 BC cases. Our IHC analysis revealed cytosolic expression of EPHX2 protein in tumor cells (Figure 1a). Interestingly, we also observed a strong nuclear expression of EPHX2 (Figure 1b). Nuclear expression was more pronounced in tumor cells, but we also noted expression by infiltrating inflammatory cells (Figure 1c).

**Figure 2:**
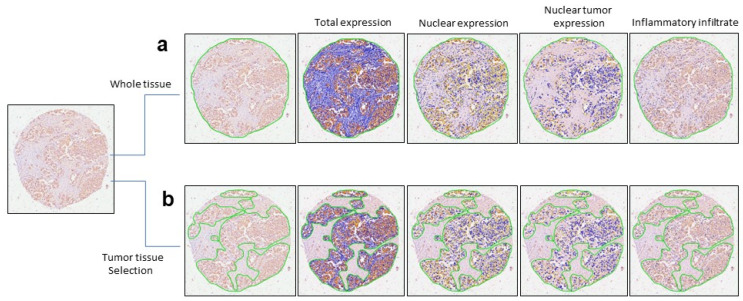
Expression and analysis of EPHX2 through digital pathology in tissue microarray from breast cancer patients. (**a**) EPHX2 expression was evaluated in the entire tissue or total area (delimited by green line) of breast cancer patients and analyzed using DP. Differential analysis included total expression, nuclear expression, nuclear expression in the tumor tissue, and the presence of inflammatory infiltration. (**b**) EPHX2 expression was assessed specifically in the tumor tissue area (delimited by green line) of breast cancer patients. After selecting this region, the analysis included total expression, nuclear expression, nuclear expression in tumor tissue, and the presence of inflammatory infiltrate. Magnification 10X.

Initially, we assessed nuclear expression throughout the entire tissue, including TMA spots (Figure 2a). However, we did not observe significance in AUC or *p*-values for these variables; therefore, these were not selected as candidate biomarkers (Supplementary Figure S2). Due to the lack of significant findings using entire tissue, we then focused on tumor areas within each TMA spot. Assisted by two expert pathologists and DP, we accurately delineated tumor areas (Figure 2b). 

### 2.3. Color Markup Image of Nuclear Staining Intensity and Biomarker Cutoff

**Figure 3:**
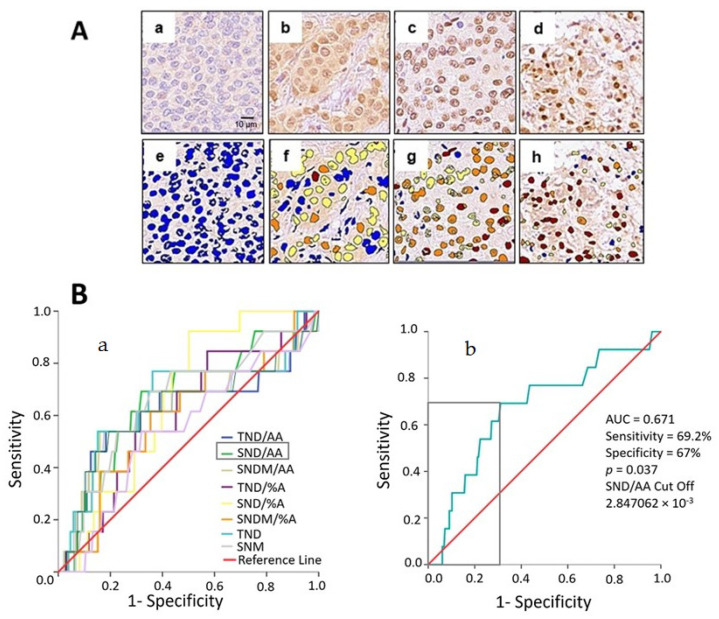
Examples of immunohistochemical and digital analysis images showing EPHX2 nuclear expression in breast cancer tissues and computer image analysis: (**A**) Differential immune stanning: (**a**) negative EPHX2 expression shown in blue (IHC) and corresponding digital analysis output (**e**), (**b**) weak EPHX2 expression depicted in yellow (IHC) and corresponding digital analysis output (**f**), (**c**) medium EPHX2 expression displayed in orange (IHC) and corresponding digital analysis output (**g**), (**d**) strong EPHX2 expression indicated in red (IHC) and corresponding digital analysis output (**h**) (scale bar = 10 μm); (**B**) (**a**) multiple ROC curves for variables of EPHX2 quantification via DP. The different colored lines represented each variable measured by DP. (**b**) selection of biomarker cutoff to optimize sensitivity and specificity. The gray box locates the optimal cutoff point.

In Figure 3, we present a representative color-coded image based on different grades of EPHX2 nuclear staining intensity observed in the delineated tumor area (Figure 3A). Grades include negative expression in blue (Figure 3A(a,e)), low expression in yellow (Figure 3A(b,f)), medium expression in orange (Figure 3A(c,g)), and strong expression in red (Figure 3A(d,h)). Using these EPHX2 expression levels, we constructed receiver operator characteristics (ROC) curves to quantify the specificity and sensitivity of multiple variables obtained from DP.

Receiver operating characteristics (ROC) curves were OS-based, considering area under the curve (AUC) and statistical significance (*p*-value) (Figure 3B(a)). Among the variables, we identified strong nuclear density per square micrometer (SND/AA) as the potential biomarker (AUC = 0.671, *p* = 0.037). To optimize the cutoff value, maximizing sensitivity and specificity, we utilized X-tile software analysis in conjunction with the ROC curves. The optimum cutoff point for SND/AA was 2.847062 × 10^−3^ pixels, resulting in a sensitivity of 69.2% and specificity of 67% (Figure 3B(b)).

### 2.4. Uni- and Multivariate Survival Analysis

**Figure 4:**
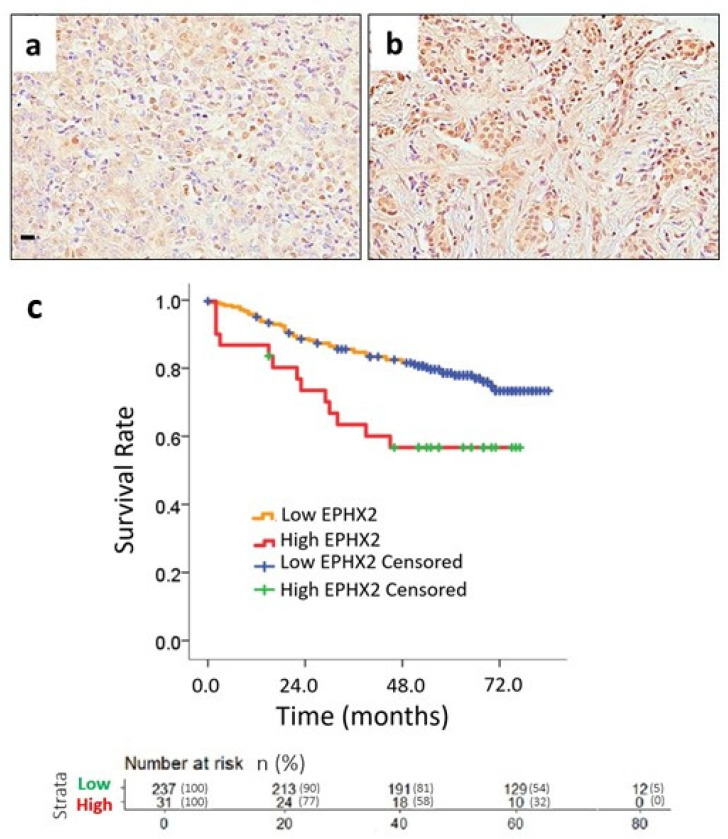
Survival curves and their correlation with EPHX2 expression in breast cancer patients: (**a**) low expression of EPHX2 via IHC staining in tumor tissue, scale bar = 10 μm; (**b**) high expression of EPHX2 via IHC staining; (**c**) Kaplan–Meier curves showing that increased EPHX2 expression is directly associated with worse prognosis, i.e., decreased OS at 52.6 months (red line); conversely, lower EPHX2 expression is associated with increased OS in BC patients, with a mean survival of 71.1 months (orange line) (median OS not reached).

Then, we explored whether EPHX2 could predict overall survival (OS) based on the selected cutoff point. Patients were stratified into low and high EPHX2 groups, and Kaplan–Meier curves were generated. Survival data were then analyzed using the log-rank method. In Figure 4, we present representative microphotographs showing either low (Figure 4a) or high EPHX2 nuclear expression (Figure 4b). Our results showed high EPHX2 levels in the nucleus, associated with poor prognosis. Median OS was not reached, but mean OS was 52.6 months for high-EPHX2 compared to 71.1 months for the low-EPHX2 group (*p* = 0.005) (Figure 4c). Table 2 presents univariate OS analysis for various clinicopathological characteristics in the BC patient cohort. Different variables showed a significant impact on OS, including nuclear grade, tumor size, axillary lymph node dissection, estrogen and progesterone receptor (ER, PR) status, molecular subtype, stage, and recurrence. To identify independent predictors of OS, we performed multivariate OS analysis (Table 3). After Cox regression, few variables remained significant, including ER status, molecular subtype, stage, recurrence, and EPHX2 expression. Notably, EPHX2 expression was confirmed as an independent predictor of survival, with a hazard ratio (HR) of 3.483, a 95% confidence interval (CI) of 1.804–6.724, and *p* < 0.001. 

### 2.5. Database Analysis

**Figure 5:**
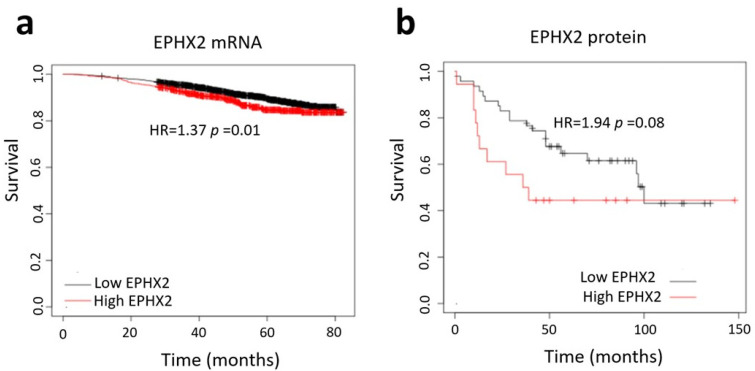
Overall survival for breast cancer based on EPHX2: mRNA (**a**) and protein data (**b**) from TCGA, GEO, and EGA databases (KM Plotter).

To study the possible relationship between EPHX2 expression and the outcomes of BC patients, we performed KM plotter analysis based on multiple genomic databases (TCGA, GEO, EGA). The results revealed that patients with elevated EPHX2 expression both at mRNA (Figure 5a) and protein levels (Figure 5b) were associated with poor prognosis.

## 3. Discussion

BC is a complex and heterogeneous disease characterized by the accumulation of genetic and epigenetic modifications in cells [19]. Despite significant progress in diagnosis and treatment, BC remains the most prevalent cancer affecting women worldwide. Therefore, there is a need to improve OS and quality of life through the search and discovery of new biomarkers.

Tumor cell activities lead to metabolic reprogramming, which is crucial for maintaining bioenergy production and redox homeostasis in the tumor [20,21]. Changes in fatty acid metabolism are among the most significant metabolic alterations in cancer. Increased lipid synthesis and uptake contributes to rapid growth of cancer cells and tumor formation [22]. Also, fatty acids play a vital role in cellular metabolism by participating in biofilm synthesis and regulating mobility [23]. While these studies have provided new insights into tumor fatty acid metabolism, the complexity of this process has not been fully understood. In this study, we examined the clinical correlation between EPHX2 and BC using a DP approach, establishing a key role for EPHX2 in BC prognosis. 

EPHX2 is expressed in various human malignant neoplasms, including prostate cancer, hepatocellular carcinoma, and colon cancer [24]. However, the published results regarding its role in cancer are still unclear. In some tumor types, such as hepatocellular carcinoma and colon cancer, low EPHX2 expression has been associated with good prognosis [25]. In the case of prostate cancer, data are controversial. Some studies indicate low EPHX2 expression correlates with favorable prognosis [26]. However, another study inhibiting EPHX2 reduced the viability of prostate cancer cells in combination with other treatments such as androgen deprivation or induction of oxidative stress [27]. These findings indicate a positive correlation between EPHX2 and prostate cancer progression. 

In addition, Bettaieb et al. [28] reported increased expression and activity of EPHX2 during the early phase of acute pancreatitis (AP). Importantly, EPHX2 deficiency mitigated the effects of cerulein- and arginine-induced AP in mice. This was associated with a reduced NF-κB inflammatory response and decreased cell death in Ephx2 knockout (KO) mice. These findings suggest a novel role for EPHX2 in the pancreas and indicate how pharmacological inhibition of EPHX2 may have therapeutic value in AP. 

The literature regarding EPHX2 expression in BC show conflicting correlations with good or bad outcomes. On the one hand, it has been demonstrated that EPHX2 are overexpressed in BC. On the other hand, Kesavan et al. [29] recently investigated the contribution of PUFA epoxides to metastasis in a spontaneous BC model. They crossed EPHX2−/− mice with the polyoma middle T oncogene (PyMT) background to examine the impact of EPHX2 deletion. The results showed that EPHX2 deletion accelerated primary tumor growth, increased tumor macrophage count, and angiogenesis. Slight differences were observed in the content of epoxide/diol compounds in tumors, particularly in epoxy octadecamonoenic acid versus dihydroxy octadecenoic acid. Additionally, significant changes were observed in the expression of proteins associated with cell proliferation and metabolism. Interestingly, EPHX2 inhibition did not affect metastasis formation in lymph nodes or lungs. These studies shed light on the involvement of EPHX2 in BC progression, providing insights into the potential therapeutic implications of targeting EPHX2 in this malignancy.

On the other hand, it is well known that arachidonic acid (AA) is metabolized through the cytochrome P450-dependent pathway that produces epoxyeicosatrienoic acids (EETs). These bioactive metabolites efficiently block inflammation and pain by inhibiting NF-κB activity. EETs conversion into dihydroxyeicosatrienoic acids (DHETs) by EPHX2 create a pro-inflammatory microenvironment [30]. Clinical significance of EPHX2 inhibition is demonstrated by existing therapeutic agents targeting this enzyme. Currently there are several studies registered in ClinicalTrials.gov retrieved using the search term “soluble hypoxide hydrolase”. Some of them use EPHX2 inhibitors such as GSK2256294, EC5026, and AR9281 to treat conditions such as insulin resistance (NCT03486223), neuroinflammation at subarachnoid hemorrhage (NCT03318783), neuropathic pain after spinal cord injury (NCT06438471), vasoconstriction in heat failure (NCT00654966), hypertension and impaired glucose tolerance (NCT00847899), and vulnerable patients such as those with obesity, smoking, older age, or depression (NCT01762774, NCT02006537, NCT05575713). There is also a study on regulation of macrophage function (NCT02743468).

As a significant and unexpected finding, in the present study, we discovered EPHX2 expression in tissue samples from BC patients to be predominantly localized in the nucleus (Figure 1). This observation has not been previously reported for BC or other cancer types. However, several studies have examined the subcellular distribution of EPHX2, finding cytoplasmic and peroxisomal localization of EPHX2 in different organs and cell types. For instance, studies by Mullen et al. demonstrated cytosolic and/or peroxisomal expression of EPHX2 in mouse and rat liver [31]. Enayetallah et al. also reported cytosolic and peroxisomal expression in human hepatocytes and renal proximal tubules, while observing exclusively cytosolic localization in other tissues such as pancreatic islet cells, intestinal epithelium, anterior pituitary cells, adrenal gland, endometrium, lymphoid follicles, prostate ductal epithelium, alveolar wall, and blood vessels [12,32]. Moreover, Pacifici et al. identified epoxide hydrolase activity in the nuclear fraction of human fetal and adult liver using enzymatic assays [33]. They also confirmed the nuclear localization of EPHX2 in rhesus monkey liver through morphological characterization [34]. Additionally, Rawal et al. reported nuclear localization of EPHX2 in neonatal rat brain cortical astrocytes using cell fractionation techniques and morphological assays [35]. These studies collectively demonstrate that EPHX2 can exhibit both cytoplasmic and peroxisomal localization across multiple tissues and cell types, and nuclear localization has been reported in specific contexts. While cytoplasmic expression of EPHX2 has been predominantly described in various tumor types [24], there have been no previous reports of nuclear expression in cancerous tissues. The identification of nuclear EPHX2 expression in primary BC tumor samples in the present study represents the first evidence of this phenomenon that warrants further investigation.

There is no information available on subcellular localization or the activity of EPHX2 in the nucleus. However, we believe that EPHX2 conversion of metabolites from linolenic acid (LA) may exert tumorigenic activity at nuclear level. Epoxidation of LA release epoxy octadecenoic acids (EpOMEs), which, in contrast to EET, are cytotoxic, associated with multiple organ damage and stimulating NF-KB activity. EPHX2 converts EpOME into more potent and toxic compounds, i.e., dihydroxyoctadecamoic compounds (DiHOMEs), inducing oxidative stress [36]. Elevated nuclear activity of EPHX2 would increase DiHOMEs and may cause genetic damage at the DNA and histone levels. This could result in genomic instability, mutational burden, and a more aggressive tumor phenotype. In addition, this agrees with our present study, where EPHX2 overexpression significantly correlated with TNBC, a heterogeneous and aggressive form of breast cancer, lacking therapeutic targets and with poor prognosis [37]. Moreover, recent evidence shows the estrogen-mediated downregulation of sEH expression via an epigenetic mechanism, i.e., the methylation of the EPHX2 gene, consistent with our observations of elevated EPHX2 levels in TNBC [38]. However, more studies are needed to elucidate the potential role of sEH in the cell nucleus.

To further analyze the significance of nuclear expression, we evaluated the whole tissue, including TMA spots (Figure 2a), without finding any significant results in ROC curve analysis for these variables; i.e., AUC and *p*-values were not relevant (Supplementary Figure S2). Therefore, we focused on tumor areas within each TMA spot, aided by two expert pathologists and DP tools for delineation (Figure 2b). With this approach, we identified significant AUC and *p*-values in ROC curves and associations between EPHX2 expression and relevant clinicopathological characteristics influencing the outcome of BC patients included in this study (Supplementary Figure S1).

DP is gaining worldwide momentum as an innovative technology associated with the improved evaluation of potential biomarker expression in cancer. DP offers the opportunity to assist in cancer diagnosis and research by enabling easy measurement, pinpointing, and selection of the relevant areas for detailed analysis of biomarker expression, thereby facilitating various research tasks [39]. The use of DP in pathology has clear potential, as demonstrated by the recent approval by the US Food and Drug Administration (FDA) of a whole slide imaging system for DP in BCr prognostics (https://www.fda.gov/news-events/press-announcements/fda-allows-marketing-first-whole-slide-imaging-system-digital-pathology; accessed 6 June 2017). In this study, we faced a challenge upon analyzing EPHX2 expression in TMA from BC within the entire tissue, without finding any significant correlation between EPHX2 expression and different clinical data. However, when two expert pathologists, aided by DP, selected and analyzed only the tumor areas (Figure 2), we observed significant association between clinical data and EPHX2 expression, as presented in this study. This result underscores DP as a valuable tool for evaluating biomarker expression in cancer. We recommend this strategy to overcome future challenges and take advantage of the opportunities offered by DP.

As mentioned above, few studies have evaluated EPHX2 expression and/or function in BC. Yue et al. [14] identified EPHX2 among seven novel genes associated with BC and developed an expression signature with significant prognostic value for relapse-free survival. However, further research is needed to fully understand the mechanisms and efficacy of EPHX2 inhibition as a therapeutic target in BC. 

Although the beneficial effects of EPHX2 inhibitors on cancer patients have not been described yet, studies in animal models conducted by Dr. Hammock’s group have shown promising results. In one study, co-administration of 19,20-epoxydocosapentaenoic acid (19,20-EDP) and trans-4-[4-(3-adamantan-1-yl-ureido)-cyclohexyloxy]-benzoic acid (t-AUCB) inhibited primary tumor growth and angiogenesis in a syngeneic Met-1 tumor model (triple-negative breast cancer), as well as metastasis in a Lewis lung carcinoma (LLC) model [40]. Subsequently, the same group demonstrated that dual inhibition of cyclooxygenase-2 and EPHX2 with celecoxib and t-AUCB, respectively, synergistically suppressed primary tumor growth and metastasis by inhibiting tumor angiogenesis [41]. Additionally, our own research has shown that the accumulation of epoxides resulting from the co-administration of 2,3,7,8-tetrachlorodibenzo-p-dioxin (TCDD) and 1-trifluoromethoxyphenyl-3-(1-propionylpiperidin-4-yl) urea (TPPU) reduced the growth and metastasis of LLC-derived tumors in mice fed a diet rich in omega-3 PUFAs, while a diet rich in omega-6 PUFAs produced the opposite effects, supporting the importance of a balanced PUFAs intake [42]. Remarkably, there are no clinical trials on EPHX2 focused on cancer, despite its potential role in inflammation-driven tumorigenesis. However, a recent study reveals that pharmacological inhibition of EPHX2, as well as dietary w-3 PUFA supplementation in different murine cancer models, can improve cancer immunotherapy against immune checkpoints such as PD-1 and CTLA [43]. Results obtained in the present study support the notion that sEH offer a potential new therapeutic strategy for BC.

## 4. Material and Methods

### 4.1. Patient Selection

In the present retrospective observational cross-sectional study, 267 clinical specimens were collected from Mexican women with pathologically confirmed diagnosis of primary BC. The cohort included patients diagnosed between 2015 and 2017 through medical records from the “Hospital General Regional No. 1”, “Instituto Mexicano del Seguro Social de Morelia”, and “Hospital General Dr. Miguel Silva” (Morelia, Michocan, México). Clinical information and tissue samples were collected under the authorization of the Ethical and Research Committees (R-2020-1602-014). OS was measured from the date of histopathological diagnosis to the date of the patient’s last follow-up or death.

### 4.2. Tissue Microarray

After identification of representative areas of tissue by two expert pathologists, the TMA was constructed using a Chemicon Advanced Tissue Arrayer (ATA 100, Sigma Aldrich, Burlington, MA, USA). Briefly, a receptor tissue block was created from a paraffin-embedded donor block, where 3 mm high cylinders were cut by a 0.5 mm needle to generate a 12 × 6 matrix. Subsequently, 3 mm high cylinders were cut from the tissues analyzed and selected for this study using a 0.4 mm needle, and each cylinder was placed in the receptor block until the matrix was completed. TMA included three cores (spots) for each patient. Finally, the block was covered with liquid paraffin and placed in an oven at 60 °C for 15 min. Once the block was cooled, 4 μm thin sections were made with a rotating microtome [42,44].

### 4.3. Immunohistochemical Analysis

IHC staining was performed manually. Prior to staining, TMA sections were baked at 60 °C for 20 min. Sections were dewaxed in xilol and rehydrated through a graded alcohol series. Antigen retrieval was performed by heating sections under pressure in citrate buffer pH 6. Endogenous peroxidases were blocked in 10% H_2_O_2_ and non-specific binding was blocked by incubating sections in 2% swine serum for 60 min at room temperature in a humidity chamber in a shaker. The EPHX2 antibody (Invitrogen, Waltham, MA, USA. Cat PA5-3351) was added and incubated overnight at 4 °C. After a PBS 1× wash, the secondary antibody from Vector Laboratories (Newark, CA, USA) was applied to sections for 30 min. After washing, sections were incubated with the ImmPRESS HRP Horse Anti-Rabbit IgG Polymer Detection Kit Peroxidase from Vector Laboratories and a DAB detection system (Vector Laboratories), counterstained with hematoxylin, dehydrated, and mounted. 

### 4.4. Digital Pathology Analysis and Automated Image Quantification

For the retrospective review via digital image analysis, an EPHX2-stained IHC slide for each case was scanned at 20× using an Aperio ScanScope CS2 whole-slide scanner (Leica Biosystems, Buffalo Grove, IL, USA). These were converted to digital images and stored on a password-protected database using Aperio eSlide Manager (version 12.3, Leica Biosystems). In these virtual slides, tumor areas were delineated by two expert pathologists. EPHX2 stained slides were annotated using the Aperio ImageScope viewing software (version 12.3, Leica Biosystems). The ScanScope generated true color digital images of each stained sample, which were viewed using Aperio ImageScope (version 6.25) software. Annotations on each EPHX2 stained slide were analyzed using the Aperio Nuclear algorithm (version 9.2, Leica Biosystems) without modifications. Default settings for the nuclear algorithm were used (“Nuclear Algorithm, User’s Guide” Leica Biosystems, MAN-0338, Revision 8; 5 August 2015) to calculate staining intensity and percent of labeled target for each sample by digitally analyzing color intensity. A color markup image for each slide was obtained based on nuclear staining intensity, and inputs were previously preconfigured for quantification of brown color, with the following thresholds: [min nuclear size (μm^2^) = 10; max nuclear size (μm^2^) = 1000; min roundness = 0.01; min compactness = 0; min elongation = 0.4; weak (1+) threshold = 220; moderate (2+) threshold = 210; strong (3+) threshold = 195]. The algorithms allow for quantification of color saturation and identify positive pixel intensity, classified as weak (yellow), moderate (orange), strong (red), and negative (blue). The output was viewed as staining intensities ranging from 0 to 3, and statistical analyses were performed using mean values. Data are presented as nuclear intensity/µm^2^ [44].

### 4.5. Database Search

The association between EPHX2 expression levels and prognosis of BC patients was analyzed via the Kaplan–Meier plotter (http://kmplot.com/analysis/) [45] (last accession: 15 June 2023). This web tool is based on multiple genomic databases including, The Cancer Genome Atlas (TCGA), Gene Expression Omnibus (GEO), and European Genome-Phenome Archive (EGA). A total of 2976 patients were analyzed for mRNA expression (RNA-seq data) and 65 for protein expression, and they were stratified according to the upper quartile of EPHX2 expression level. The two groups were contrasted to evaluate OS without restrictions on status for clinicopathological characteristics such as lymph node dissection, estrogen, progesterone and HER2 receptors, KI67 level, Nottingham histologic grade, or PAM50 subtype, including endocrine treatment and chemotherapy.

### 4.6. Statistical Analysis

Data were analyzed using SPSS Statistics for Windows, Version 25.0 (IBM Corp., Armonk, NY, USA, 2017). Results were reported as mean ± standard deviation or median and range for normal and non-normal variable distributions, respectively. Normality was evaluated via the Kolmogorov–Smirnov test (n > 50). The receiver operator characteristics (ROC) curve, area under the curve (AUC), and X-tile software (version 3.6.1) [46] were used for the optimization of variables and the cutoff points for EPHX2 as the biomarker. The relationship between EPHX2 expression and different clinicopathological variables was assessed via Chi-squared and Fisher’s exact tests for categorical variables and Mann–Whitney U and Student´s *t*-tests for continuous data. Univariate and multivariate analyses were based on the Cox proportional hazards model to identify variables with a prognostic value for OS. Survival curves and risk tables were generated by the R software (version 4.4.1) and the Kaplan–Meier method, whereas differences between high and low expression groups were calculated by the Log Rank test. Statistically significant results were indicated by *p* < 0.05.

## 5. Conclusions

In conclusion, our work is the first study describing the nuclear expression of EPHX2 in breast tumors (BC). This study emphasizes the upregulation of the nuclear expression of EPHX2 as a predictive factor for poor outcomes in BC patients. The application of a digital pathology approach was instrumental in identifying this significant association. Our findings offer valuable insights into the potential clinical relevance of EPHX2 as a prognostic biomarker in BC.

## Figures and Tables

**Table 1:** Clinicopathological characteristics and EPHX2 expression in BCA patients.

Variable n (%)	N	EPHX2 Low (n = 234)	EPHX2 High (n = 33)	Total (n = 267)	*p*-Value
Age	267				0.710 ^C^
<52 yr		111 (47.4%)	14 (42.4%)	125 (46.8%)	
≥52 yr		123 (52.6%)	19 (57.6%)	142 (53.2%)	
Histologic Grade	265				0.751 ^F^
I		46 (19.8%)	8 (24.2%)	54 (20.4%)	
II		124 (53.4%)	18 (54.5%)	142 (53.6%)	
III		62 (26.7%)	7 (21.2%)	69 (26.0%)	
Nuclear Grade	265				0.505 ^F^
1		19 (8.2%)	4 (12.1%)	23 (8.7%)	
2		145(62.5%)	17 (51.5%)	162 (61.1%)	
3		68 (29.3%)	12 (36.4%)	80 (30.2%)	
Border Type	267				0.030 ^F^*
Non-		172 (73.5%)	30 (90.9%)	202 (75.7%)	
Infiltrative		62 (26.5%)	3 (9.1%)	65 (24.3%)	
Tumor Size	267				0.090 ^C^*
≤2 cm		94 (40.2%)	19 (57.6%)	113 (42.3%)	
>2 cm		140 (59.8%)	14 (42.4%)	154 (57.7%)	
ALND	267				1.000 ^C^
No		84 (35.9%)	12 (36.4%)	96 (36.0%)	
Yes		150 (64.1%)	21 (63.6%)	171 (64.0%)	
ER Status	267				0.109 ^C^
Negative		70 (29.9%)	15 (45.5%)	85 (31.8%)	
Positive		164 (70.1%)	18 (54.5%)	182 (68.2%)	
PR Status	267				1.000 ^C^
Negative		98 (41.9%)	14 (42.4%)	112 (41.9%)	
Positive		136 (58.1%)	19 (57.6%)	155 (58.1%)	
HER2 Status	267				1.000 ^C^
Negative		192 (82.8%)	27 (81.8%)	219 (82.6%)	
Positive		40 (17.2%)	6 (18.2%)	46 (17.4%)	
Molecular Subtype	260				0.330 ^F^
LUM A + B		163 (71.2%)	18 (58.1%)	181 (69.6%)	
HER2		23 (10.0%)	4 (12.9%)	27 (10.4%)	
TNBC		43 (18.8%)	9 (29.0%)	52 (20.0%)	
AJCC-PS	264				0.323 ^F^
I		86 (37.2%)	12 (36.4%)	98 (37.1%)	
II		90 (39.0%)	10 (30.3%)	100 (37.9%)	
III		51 (22.1%)	9 (27.3%)	60 (22.7%)	
IV		4 (1.7%)	2 (6.1%)	6 (2.3%)	
Recurrence	267				0.506 ^C^
No		183 (78.2%)	24 (72.7%)	207 (77.5%)	
Yes		51 (21.8%)	9 (27.3%)	60 (22.5%)	

^C^ Chi-square test; ^F^ Fisher’s exact test. ALND: axillary lymph node dissection; ER: estrogen receptor; PR: progesterone receptor; HER2: human epidermal growth factor receptor 2; LUM: luminal; TNBC: triple-negative breast cancer; AJCC: American Joint Committee on Cancer; PS: prognostic stage. * *p* < 0.05.

**Table 2:** Univariate analysis of survival in breast cancer patients (Cox regression).

		Univariate Analysis		
Variable	Category	HR	95% CI	*p*-Value
Age (Years)	≥52 vs. <52	1.100	0.689–1.755	0.690
Histologic Grade	III vs. I + II	1.262	0.750–2.123	0.381
Nuclear Grade	3 vs. 1 + 2	1.823	1.127–2.948	0.014 *
Border Type	Infiltrative vs. Non	1.333	0.742–2.393	0.336
Tumor Size	>2 cm vs. ≤2	1.638	1.002–2.680	0.049 *
ALND	Yes vs. No	0.481	0.302–0.766	0.002 *
ER Status	+ vs. −	0.558	0.348–0.894	0.015 *
PR Status	+ vs. −	0.561	0.352–0.894	0.015 *
HER2 Status	+ vs. −	0.894	0.469–1.701	0.732
Molecular Subtype	TNBC vs. LUM A + B	1.815	1.055–3.123	0.031 *
	HER2 vs. LUM A + B	2.054	1.029–4.097	0.041 *
AJCC Prognostic Stage	II vs. I	2.281	1.172–4.441	0.015 *
	III vs. I	5.778	2.987–11.180	<0.001 *
	IV vs. I	5.216	1.173–23.189	0.030 *
Recurrence	Yes vs. No	3.939	2.465–6.294	<0.001 *
EPHX2	High vs. Low	2.316	1.261–4.252	0.007 *

HR: hazard Ratio; 95% CI: Confidence Interval; ALND: axillary lymph node dissection; ER: estrogen receptor; PR: progesterone receptor; HER2: human epidermal growth factor receptor 2; LUM: luminal; TNBC: triple-negative breast cancer; AJCC: American Joint Committee on Cancer. * *p* < 0.05.

**Table 3:** Multivariate analysis of survival in breast cancer patients (Cox regression).

		**Multivariate Analysis**		
**Variable**	**Category**	**HR**	**95% CI**	***p*-Value**
Nuclear Grade	3 vs. 1 + 2	1.412	0.801–2.487	0.232
Tumor Size	>2 cm vs. ≤2	1.399	0.734–2.665	0.308
ALND	Yes vs. No	0.629	0.284–1.392	0.253
ALND (Number)	≥10 vs. <10	0.723	0.318–1.645	0.439
ER Status	+ vs. −	0.041	0.005–0.357	0.004 *
PR Status	+ vs. −	0.772	0.328–1.816	0.553
Molecular Subtype	TNBC vs. LUM	0.712	0.305–1.661	0.432
	HER2 vs. LUM	14.855	1.446–152.623	0.023 *
AJCC Prognostic Stage	II vs. I	2.008	0.904–4.458	0.087
	III vs. I	4.295	1.719–10.731	0.002 *
	IV vs. I	1.375	0.243–7.786	0.719
Recurrence	Yes vs. No	3.714	2.167–6.365	<0.001 *
EPHX2	High vs. Low	3.483	1.804–6.724	<0.001 *

HR: hazard Ratio; 95% CI: Confidence Interval; ALND: axillary lymph node dissection; ER: estrogen receptor; PR: progesterone receptor; HER2: human epidermal growth factor receptor 2; LUM: luminal; TNBC: triple-negative breast cancer; AJCC: American Joint Committee on Cancer. * *p* < 0.05.

## Data Availability

Datasets analyzed for this study can be found in the Kaplan–Meier plotter (http://kmplot.com/analysis/) (last accessed on 30 June 2023).

## Supplementary Materials

The following supporting information can be downloaded at https://www.mdpi.com/article/10.3390/ijms25158024/s1.
