# Supplementary material for: Upregulated Nuclear Expression of Soluble Epoxide Hydrolase Predicts Poor Outcome in Breast Cancer Patients: Importance of the Digital Pathology Approach
Source: Int J Mol Sci. 2024 Jul 23;25(15):8024. doi: 10.3390/ijms25158024 (PMC11312095; doi:10.3390/ijms25158024)
Supplement: Supplementary file 1 [file ijms-25-08024-s001.zip › ijms-3087536-supplementary.pdf]

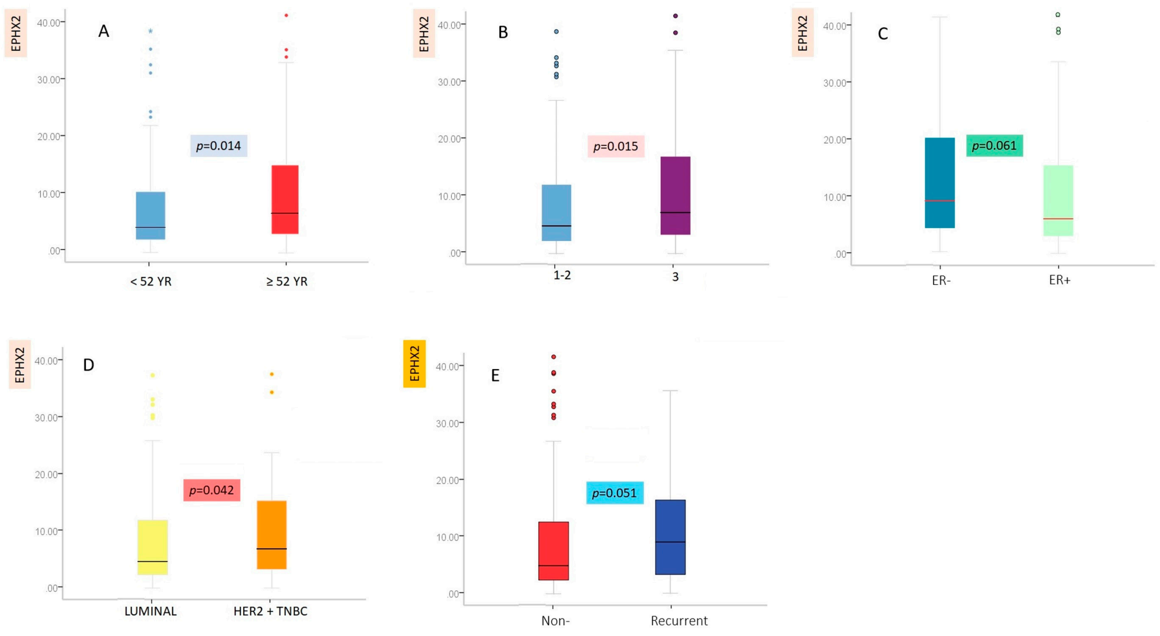

Supplementary Figure S1. EPHX2 protein expression relationship with clinicopathological characteristics of breast cancer patients: A) Age B) Nuclear Grade C) Estrogen Receptor D) Subtype E) Recurrence

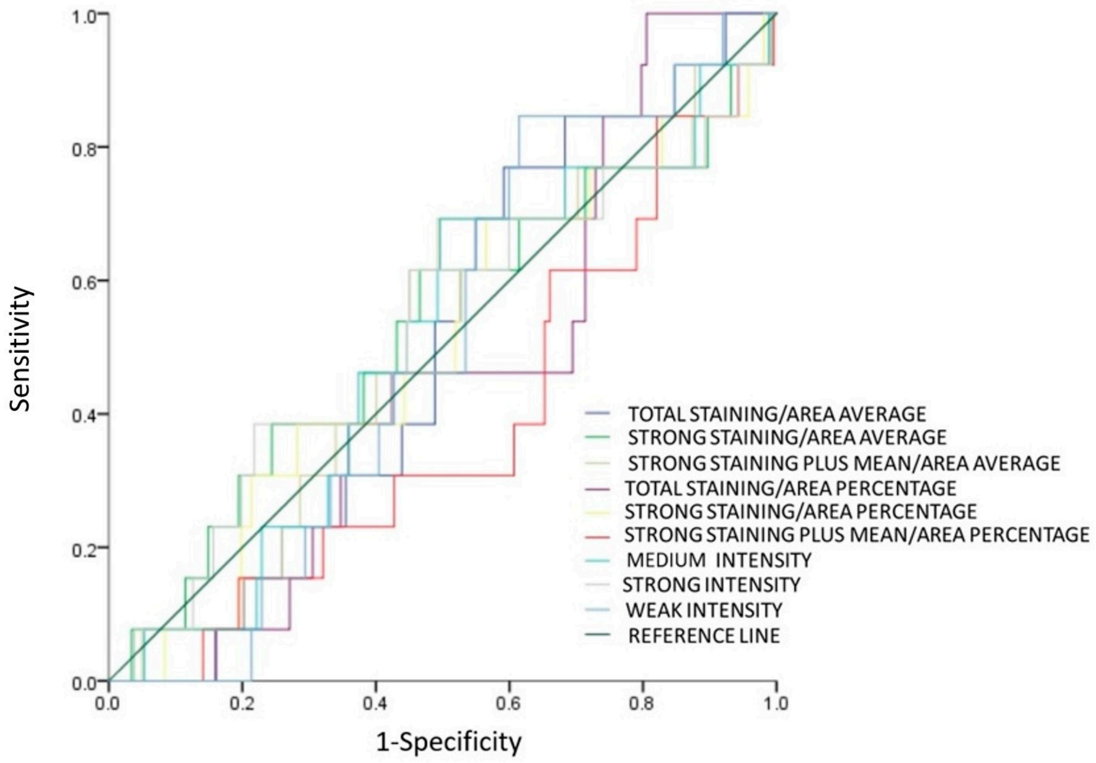

Variables	Area	P value	95% Confidence Interval Lower Limit	Upper Limit
TOTAL STAINING/AREA AVERAGE	0.499	0.989	0.371	0.627
STRONG STAINING/AREA AVERAGE	0.526	0.753	0.351	0.701
STRONG STAINING PLUE MEAN/AREA AVERAGE	0.510	0.903	0.355	0.665
TOTAL STAINING/AREA PERCENTAGE	0.457	0.599	0.327	0.586
STRONG STAINING/AREA PERCENTAGE	0.499	0.989	0.337	0.661
STRONG STAINING PLUE MEAN/AREA PERCENTAGE	0.383	0.153	0.235	0.530
IMEDIUM INTENSITY	0.505	0.952	0.351	0.659
STRONG INTENSITY	0.521	0.797	0.346	0.697
WEAK INTENSITY	0.500	1.000	0.378	0.622

Supplementary Figure S2. ROC curve analysis of EPHX2 protein by digital pathology of whole spot *i.e.* normal + tumor area
